# High-efficiency 14-plex cSNP SNaPshot assay for forensic peripheral blood identification

**DOI:** 10.1186/s12864-026-13173-0

**Published:** 2026-07-11

**Authors:** Yixia Zhao, Yuting Sun, Siyi Wei, Qinglin Liu, Anquan Ji, Caixia Li, Bofeng Zhu

**Affiliations:** 1 Institute of Evidence Law and Forensic Science, China University of Political Science and Law, Beijing, 100088 China; 2 MPS’s Key Laboratory of Forensic Genetics, National Engineering Laboratory for Crime Scene Evidence Investigation and Examination, Institute of Forensic Science, Ministry of Public Security (MPS), Beijing, 100038 China; 3 Guangzhou Key Laboratory of Forensic Multi-Omics for Precision Identification, School of Forensic Medicine, Southern Medical University, Guangzhou, Guangdong 510515 China; 4 School of Forensic Medicine, Shanxi Medical University, Jinzhong, Shanxi 030619 China

**Keywords:** Forensic peripheral blood identification, mRNA, cSNPs, SNaPshot assay

## Abstract

The analysis of body fluid stains found at criminal scenes requires not only DNA profiles but also identification of the composition of these stains, particularly in mixed stains. Prior studies have demonstrated that forensic body fluids can be differentiated by their mRNA biomarkers.In this study, we screened a panel including 14 cSNPs located on four peripheral blood mRNA biomarkers (*CD3G*, *ANK1*, *SPTB*, and *GYPA*) and developed a multiplex assay for forensic blood identification using the SNaPshot method. Particularly, a one-step multiplex reverse transcription PCR (RT-PCR) strategy was used to generate cDNA amplicons from the corresponding mRNA by reverse transcription. The sensitivity, specificity, and capability of the multiplex SNaPshot assay for blood identification were systematically assessed. These results showed that the panel of 14 cSNPs successfully realized peripheral blood identification, including single-source body fluid as well as mixed samples. However, several peripheral blood cSNP markers were detected at low expression levels in menstrual blood and vaginal secretions. The detection sensitivity of the multiplex assay reached 1 ng total RNA input. Furthermore, 14 cSNP loci were genotyped across 19 peripheral blood samples, yielding 100% concordance between DNA- and RNA-derived profiles. Using allele frequencies of 14 cSNP loci in 100 unrelated Han individuals from northern China, the cumulative discrimination power of the 14 cSNPs was calculated to be 0.98303.

**Supplementary Information:**

The online version contains supplementary material available at https://doi.org/10.1186/s12864-026-13173-0.

## Background

In forensic genetic analysis, single nucleotide polymorphisms (SNPs) exhibit high genome-wide distribution density, and short PCR amplicons (<100 bp). They address the drawback traditional short tandem repeat (STR) analysis for trace and degraded biological samples and serve as powerful markers for individuals identification, as well as lineage, ancestry and phenotype inference [1, 2]. With the improvement in DNA detection capability, the success rate of DNA analysis for even extremely trace amounts in samples has significantly improved. This progress has elevated DNA transfer and activity levels to the status of “advanced” topics in the interpretation of forensic genetic evidence [3, 4].

Body fluid stains are commonly used biological samples for DNA profiling in crime scene investigations. Since 2010, the European DNA Profiling Group (EDNAP) has organized multiple forensic laboratories to evaluate the feasibility of mRNA analysis for identifying forensic samples of peripheral blood, menstrual blood, semen, saliva, vaginal secretions, and skin using capillary electrophoresis (CE) platforms, as well as the Illumina MiSeq/FGx and the Ion Torrent PGM/S5 platforms [5–10]. DNA/RNA co-extraction technology [11, 12] and the highly integrated next-generation forensic DNA/RNA sequencing workflow enable the simultaneous acquisition of DNA and RNA profiles from a single stain sample [13]. This allows for concurrent individual identification and body fluid origin tracing, minimizing sample consumption and avoiding interpretive biases caused by positional differences in traditional “stepwise sampling.”

**Fig. 1:**
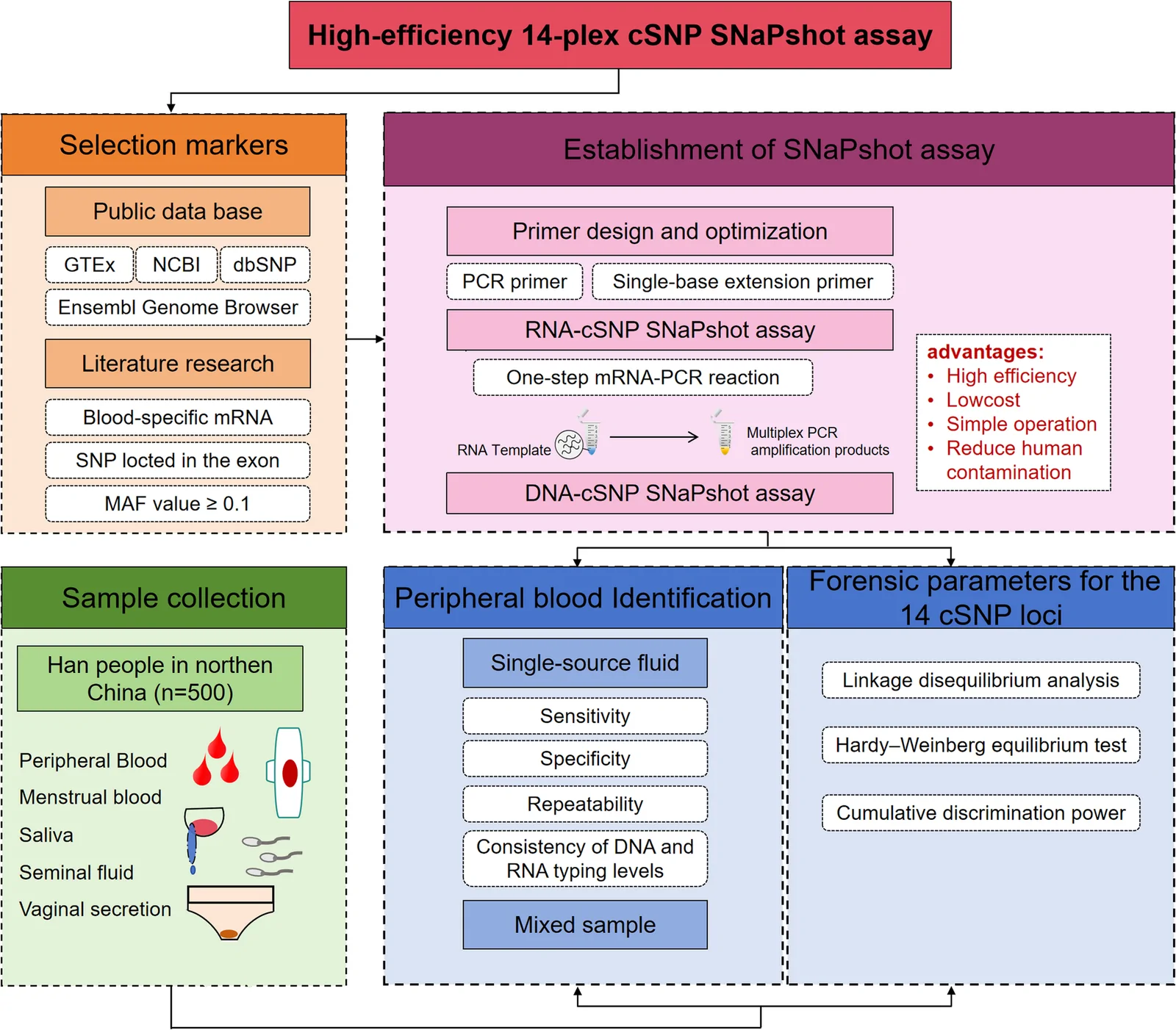
Schematic overview of the high-efficiency 14-plex coding-region single nucleotide polymorphism (cSNP) SNaPshot assay, including selection markers, sample collection, establishment of the SNaPshot assay, peripheral blood identification and forensic parameters validation

SNPs in the coding regions of the genome, known as cSNPs, can simultaneously transmit polymorphic information into mRNA molecules during DNA transcription and further determine the structure, function, and activity of proteins or enzymes [14, 15]. Previous studies have verified the cSNP is located within the body fluid mRNA transcripts. Based on extensive literature research and data mining in the Ensembl Genome Browser, we validated primers of candidate genes via both in-silico analysis and wet lab experiments and 14 cSNP loci from 4 peripheral blood genes were identified. We successfully constructed a multiplex SNaPshot minisequencing assay and evaluated it’s potential applications in distinguishing peripheral blood from mixed samples. Furthermore, this study optimized the SNaPshot workflow by introducing one-step multiplex RT-PCR before single-base extension, and compared its amplification efficiency with the traditional two-step method using mixed body fluid samples (Fig. 1). Our results demonstrated that 14 cSNPs loci derived from peripheral blood mRNA markers provide a promising strategy for blood component discrimination.

## Methods

### Sample collection and storage

All samples were collected with full informed consent from participants and approval from the Ethics Committee of the Institution of Forensic Science, Ministry of Public Security (reference #Scientific Research Ethics Approval Document of the Institute of Forensic Science of China). Body fluid samples were collected from 200 unrelated healthy Han Chinese individuals aged 18–35 years in northern China (100 males and 100 females). The sample set included 100 semen samples from male donors and 100 menstrual blood samples and 100 vaginal secretion samples from female donors, as well as 100 peripheral blood samples and 100 saliva samples randomly collected regardless of gender. Peripheral blood was collected by venipuncture into ethylenediaminetetraacetic acid–containing tubes. Menstrual blood samples (on day 2 or 3 of menstruation) were taken by the volunteers themselves using tampons. Vaginal secretions were collected at least 3 days after menstruation using sterile medical swabs. Saliva (fasting for 1 h before sample collection) was collected using sterile 2 mL Corning polyethylene tubes to collect naturally occurring oral fluids. The freshly ejaculated semen was placed in a sterile container. Swabs and tampons were air-dried at 26℃ for 24 h and stored at − 80 °C, whereas the other types of samples were collected and immediately stored at − 80 °C before RNA extraction.

### cSNP selection and primer design

The peripheral blood mRNA biomarkers, as well as positive control markers for menstrual blood, semen, saliva, and vaginal secretions, were obtained as described in the literature [5, 6, 16–19] and from the GTEx database (https://www.gtexportal.org/home/). We first identified 11 peripheral blood mRNA marker genes (SPTB, CD3G, PRF1, HBB, AMICA1, ANK1, HBA, PBGD, CD93, ALAS2, and GYPA). A total of 45 cSNPs with a minor allele frequency (MAF) value ≥ 0.10 were identified in the exon regions of peripheral blood genes using East Asian population data from the 1000 Genomes Project, accessed via the Ensembl Genome Browser (https://www.asia.ensembl.org/) (Table S1). Primer sets were designed using Primer Premier v5.0. To avoid cross-interference between DNA and RNA templates: for the RNA-cSNP assay, the mRNA-specific PCR amplification primers were required to overlap exon-exon junctions or span at least one intron. For the DNA-cSNP assay, at least one of the forward or reverse DNA multiplex PCR primers was required to be located within an intron. Corresponding amplification primers targeting genomic DNA (gDNA) were designed for all sites. Single-base extension (SBE) primers were designed with the 3′-terminal nucleotide immediately adjacent to each target SNP. Variable-length poly (CT) tails were appended to the 5′ end of each primer to generate fragment spacing of ≥ 5 nt within the same fluorescent dye channel. AutoDimer software was employed to evaluate the potential cross-binding and primer-dimer formation of both SBE primers and amplification primers. Melting temperature (Tm, nearest-neighbor algorithm) and GC content of only the core annealing region (5′ poly-CT tails excluded from parameter calculation) were calculated via the IDT online OligoAnalyzer tool (https://sg.idtdna.com). Design thresholds were restricted to a GC range of 40%–55% and a Tm window of 53–60 °C, with inter-primer Tm differences across the multiplex panel limited to ≤ 3 °C. All synthesized SBE primers were pre-tested by capillary electrophoresis to confirm fully resolved, non-overlapping genotyping peaks prior to final multiplex assay establishment. Subsequently, MFEprimer-3.1 [20] and the basic local alignment search tool from NCBI were employed to preliminarily exclude potential non-specific amplification. Agarose gel electrophoresis was performed to verify the specificity of the PCR primers, eliminating genes and primers that exhibited cross-reactivity among the five body fluids or yielded unsatisfactory amplification.

Ultimately, 14 cSNP loci from 4 peripheral bloodmarker genes (*CD3G*, *ANK1*, *SPTB*, and *GYPA*) were selected to construct a multiplex SNaPshot minisequencing system for peripheral blood, and five nucleotide sites without polymorphisms on the body fluid mRNA markers were selected as a positive control to indicate the types of body fluids, including *GYPA* for peripheral blood, *MMP7* for menstrual blood, *PRM*1 for semen, *HTN3* for saliva, and *MUC22* for vaginal secretions. Additional information about the mRNAs, cSNP sites, and positive control loci is shown in Tables 1 and 2.

**Table 1:** cSNP locus information of genes in the peripheral blood

Body fluid	Gene	SNP	Chr (GRCh38)	Allele	Minor allele frequency (MAF)
Peripheral blood (PB)	*CD3G*	rs3753058	11:118350635	A/C	0.30
		rs3753059	11:118350634	T/C	0.30
	*ANK1*	rs2304871	8:41727920	C/T	0.23
		rs1137177	8:41706167	C/T	0.18
		rs750625	8:41668396	A/G	0.19
	*SPTB*	rs229586	14:64796629	G/A	0.28
		rs229587	14:64796582	G/A	0.47
		rs77806	14:64786514	A/G	0.47
		rs1741487	14:64779244	T/C	0.41
		rs1741488	14:64779238	T/C	0.41
	*GYPA*	rs7682260	4:144120567	T/C	0.29
		rs7687256	4:144120555	A/G	0.21
		rs7658293	4:144120554	G/T	0.20
		rs4867	4:144120533	C/T	0.13

**Table 2:** Positive control locus information of genes in five body fluids

Body fluid	Gene	Chr (GRCh38)	Fixed base
Peripheral blood (PB)	*GYPA*	4:144140633	G
Menstrual blood (MB)	*MMP7*	11:102527911	G
Saliva (SA)	*HTN3*	4:70033187	A
Seminal fluid (SE)	*PRM1*	16:11281015	A
Vaginal secretion (VA)	*MUC22*	6:31032274	C

Detailed information of PCR amplicons and their length, concentrations, sequence of amplification primers, and SBE primers is presented in Tables S2 and S3. The results of the primer specificity validation of the 12 mRNA amplicons are shown in Fig. S1.

### Nucleic acid extraction and quantification

Total RNA was extracted from 500 samples of five body fluids—60 µL of peripheral blood (PB), 200 µL of semen, 500 µL of saliva, 1 cm^2^ of a tampon containing menstrual blood, and 1 sterile swab containing vaginal secretion—using an RNeasy Mini Extraction Kit (Qiagen, Germany). For DNA/RNA co-extraction, 19 randomly selected PB samples were processed using the AllPrep DNA/RNA/miRNA Universal Kit (Qiagen, Germany). Genomic DNA contamination in total RNA was eliminated using the gDNA eliminator spin column provided with the kit. Nucleic acid quantification was performed using a Qubit 4 fluorescence detector, RNA concentration was measured using the Qubit™ RNA HS Assay Kit (Invitrogen, USA), and DNA concentration was determined using the Qubit™ dsDNA HS Assay Kit (Invitrogen, USA). Meanwhile, RNA integrity was measured with the Qubit RNA Integrity & Quality (IQ) Assay Kit (Invitrogen, USA) in preliminary assay optimization experiments. Since most forensic biological samples are low-template and degraded, we did not set an RNA integrity threshold (RIN ≥ 7.0) as a mandatory sample inclusion criterion. Total RNA was directly subjected to multiplex RT-PCR or immediately stored at − 80 °C before use.

### Preparation of mixed samples from different body fluids

Three samples were randomly selected from each body fluid to prepare the mixed samples. The RNA samples were mixed in the proportions specified in Table 3. For these mixed samples, the total RNA input of PB samples was fixed at 5 ng, with the other four body fluids added in predetermined ratios, and the mixed samples were divided into multiple tubes to avoid repeated freezing and thawing. Three replicates were set up for each proportion of the mixed samples. In addition to the mixed RNA samples, a 1:1 mixed sample was prepared by combining 5 µL of PB with an equal volume of saliva.

**Table 3:** Preparation of the mixed samples

	Body fluid(s)	Mixture ratio
1	PB་SA	1:1/1:10/1:50/1:100
2	PB་SE	1:1/1:10/1:50/1:100
3	PB་VA	1:1/1:10/1:50/1:100
4	PB་SA་SE	1:25:25
5	PB་SA་VA	1:25:25
6	PB་SE་VA	1:25:25
7	PB་SA་SE་VA	1:10:30:10

### Construction of PB RNA-cSNP SNaPshot assay

The PB RNA-cSNP SNaPshot assay used RNA as the template and sequentially included multiplex RT-PCR amplification, product purification, SNaPshot SBE, and extension product repurification. Details of the PCR primers, SBE primers, and their concentrations are provided in Tables S2 and S3.

### Two-step multiplex RT-PCR amplification

cDNA was synthesized using the SuperScript™ III First-Strand Synthesis System (Invitrogen, USA). The reaction mixture contained a maximum volume of 1 µL of 2.5 mM dNTP, 1 µL of 50 µM oligo(dT), 0.1–10.0 ng RNA input, and nuclease-free water to make up the volume to 10 µL. The mixture was denatured at 65 °C for 5 min and then placed on ice for at least 1 min.

The denatured reaction mixture was amplified by adding 2 µL of 10× RT Buffer, 4 µL of 25 mM MgCl_2_, 2 µL of 0.1 M DTT, 1 µL of SuperScript™ Ⅲ reverse transcriptase (200 U/µL), 1 µL of 40 U/µL RNaseOUT™, and nuclease-free water to make up the volume to 20 µL. After the reverse transcription reaction, 1 µL of RNase H (2 U/µL) was added to digest the untranscribed RNA, yielding 21 µL of the final product. A negative control was established using nuclease-free water instead of reverse transcriptase.

The thermal cycling conditions were 50 °C for 50 min and 85 °C for 5 min, after which the reaction mixture was placed on ice, and after adding RNase H, it was incubated at 37 °C for 20 min.

The multiplex PCR reaction mixture contained 1 µL of 10× PCR buffer, 0.2 µL of 2.5 mM dNTP, 0.6 µL of 25 mM Mg^2+^, 0.1 µL of 5 U/µL Hotstar Taq enzyme (Qiagen, Germany), and 1 µL of 0.1–2.0 µM primer mixture. Nuclease-free water was added to make up the reaction volume to 10 µL.

The thermal cycling conditions were as follows: 95 °C for 15 min; 30 cycles of 94 °C for 30 s, 60 °C for 30 s, and 72 °C for 40 s; 72 °C for 10 min, and storage at 4 °C.

### One-step multiplex RT-PCR amplification

Reverse transcription and PCR amplification of the indicated amount of total RNA were carried out in a one-step reaction using the One-Step RT-PCR Kit (Suzhou Microread Genetics Co., Ltd., China). The reaction mixture contained 4 µL of 2.5× RT-qPCR buffer, 0.65 µL of RT-qPCR enzyme premix, 1 µL of 0.1–2.0 µM primer mixture, 0.1–10 ng of RNA input, and nuclease-free water to make up 10µL. For each sample, a negative control was included, for which 1 µL nuclease-free water or RT minus was added instead of the template. The thermal cycling conditions were as follows: cDNA synthesis at 55 °C for 10 min; activation of DNA polymerase at 95 °C for 5 min; 30 cycles of 95 °C for 30 s and 60 °C for 1 min; followed by a final extension at 60 °C for 30 min and storage at 4 °C.

### Amplification product purification

Purification of PCR amplification products was performed in a reaction system containing 5 µL of PCR amplification product, 0.2 µL of Exonuclease I (ExoI) (Applied Biosystems, USA), 1 µL of shrimp alkaline phosphatase (SAP; Applied Biosystems, USA), 0.3 µL of 10× SAP buffer, and 1.5 µL of ddH_2_O. The reaction was carried out at 37 °C for 30 min followed by 96 °C for 10 min, and storage at 4 °C.

### SNaPshot SBE and second purification

SNaPshot SBE and second purification were performed using the SNaPshot™ multiplex kit (Applied Biosystems, USA). The reaction system included 2.5 µL of SNaPshot multiplex reagent, 1.5 µL of purified PCR product, and 1 µL of primer. The reaction program consisted of 25 cycles of 96 °C for 10 s, 50 °C for 5 s, and 60 °C for 30 s and storage at 4 °C.

After the extension reaction, 0.5 µL SAP enzyme was added to the reaction SNaPshot system for purification. The thermal conditions were 37 °C for 60 min and 75 °C for 15 min, followed by storage at 4 °C.

### Construction of the PB DNA-cSNP SNaPshot assay

In the DNA-cSNP SNaPshot assay, gDNA was used as a template for PCR amplification using Multiplex PCR Mastermix Reagent (Polymeric Bio, China). The reaction system contained 5 µL of 2.5× Multiplex PCR Mastermix, 0.5 µL of 0.1–2.0 µM primer mixture, and 1 ng of DNA sampling volume. Nucleic acid–free water was added to make up the reaction volume to 10 µL. The thermal cycling conditions were as follows: 95 °C for 15 min; 30 cycles of 94 °C for 30 s, 60 °C for 30 s, 72 °C for 1 min; 72 °C for 10 min; and storage at 4 °C. The subsequent steps were the same as those in the RNA-cSNP assay.

### CE and profile analysis

The purified extension products were detected by CE using the ABI 3500 xL Genetic Analyzer (Applied Biosystems, USA). Mixtures of 1 µL products, 8.6 µL Hi-Di™ Formamide (Applied Biosystems, USA), and 0.4 µL Microreader™ Size Standard QD550 (Suzhou Microread Genetics Co., Ltd., China) were denatured at 95 °C for 3 min, followed by snap-cooling on ice for 5 min. The results were analyzed with GeneMapper™ ID-X software v1.5 (Applied Biosystems, USA) with a detection threshold of 100 relative fluorescence units (RFU). Peaks below 100 RFU were excluded for evaluating allelic dropout and imbalance. At heterozygous loci, a peak height ratio (minor/major peak) < 0.1 indicated severe allelic imbalance, and such genotypes were interpreted cautiously. The analysis parameters included bin sets, which include the size range and dye expected for a given fragment.

### Statistical analysis

The raw fluorescence intensity values of 14 cSNP loci were preprocessed via logarithmic transformation, followed by principal component analysis (PCA) and t-distributed stochastic neighbor embedding (t-SNE) dimensionality reduction implemented in R. Specifically, PCA was conducted using the built-in *prcomp* function, whereas t-SNE was performed using the *Rtsne* package. Subsequently, the *ggplot2* package was used to visualize the dimensionality-reduced outputs.

The fluorescence values of the 14 cSNP loci were statistically analyzed using GraphPad Prism 10.5.0. One‑way analysis of variance followed by Tukey’s multiple comparison correction was conducted, with a corrected P value ≤ 0.05 defined as statistically significant.

The STRAF online tool (straf-p7bdrhm3xq-ew.a.run.app/) was used to evaluate linkage disequilibrium, Hardy–Weinberg equilibrium (HWE) tests, and calculate forensic parameters for the 14 cSNP loci: expected heterozygosity, polymorphism information content, probability matching, discrimination power, observed heterozygosity, probability of exclusion, and typical paternity index. For loci in linkage disequilibrium, the PHASE v2.1 software was used to infer haplotypes, and then the forensic parameters were recalculated using STRAF.

## Results

### Establishment of multiplex SNaPshot assay

In this study, before system establishment, in-silico PCR experiments [21] were performed using MFEprimer-3.1 software. This analysis was conducted to evaluate the efficiency and specificity of primers for candidate mRNA markers. Subsequently, the expression specificity of these candidate markers across the five body fluids was verified via agarose gel electrophoresis. The in-silico PCR amplification and agarose gel electrophoresis products of *CD3G*, *ANK1*, and *SPTB* were all single band. For *GYPA*, one band was observed in agarose gel electrophoresis, whereas in-silico PCR yielded two fragments. Sequence alignment revealed that the sequence of the 100–250 bp product of *GYPA* completely overlapped with that of the 250–500 bp fragment, indicating that both amplification products of *GYPA* were specifically expressed in the PB (Fig. S1).

**Fig. 2:**
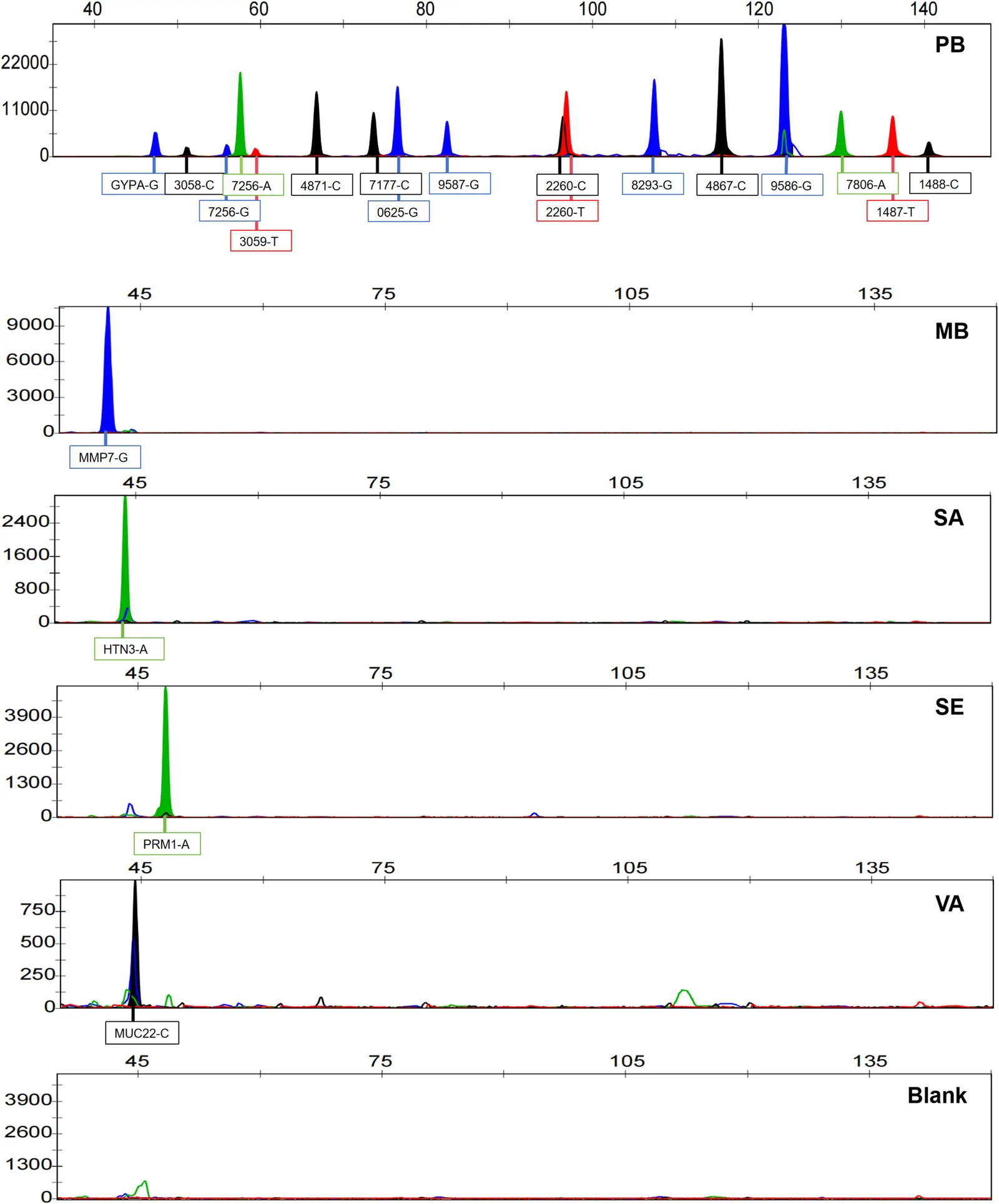
Genotyping of 14 peripheral blood coding-region single nucleotide polymorphism (cSNP) loci in five body fluids using the SNaPshot assay when RNA input was 5 ng. Each peak corresponds to the allele of a specific locus, labeled by the last four digits of its respective rs number. Nonpolymorphic nucleotide loci on genes specific to the five body fluids were used as positive controls. The fluorescence colors indicate the type of nucleotide bases: blue for G, green for A, black for C, and red for T. PB, peripheral blood; MB, menstrual blood; SA, saliva; SE, semen; VA, vaginal secretion

Fourteen cSNP loci located on 4 PB mRNA biomarkers were identified. In combination with one-step multiplex RT-PCR amplification, a multiplex SNaPshot minisequencing assay was established. Furthermore, nonpolymorphic nucleotide loci from genes specific to menstrual blood, saliva, semen, and vaginal secretions were included in the assay as positive controls, and nuclease-free water was used instead of the RNA template as a blank control. The results demonstrated that genotyping of all 14 cSNP loci was successfully detected in single PB samples. In the other 4 types of body fluids, only the corresponding positive control peaks were detected, and no peaks were detected in the blank control (Fig. 2).

### Study on the assay detection sensitivity

To evaluate the detection sensitivity of the assay, total RNA from PB at concentrations of 10, 5, 1, 0.5, and 0.1 ng was used as template. The peak heights of the 14 cSNP loci were measured under different RNA input amounts, where the fluorescence value of each locus was the sum of the fluorescence values of its two alleles.

**Fig. 3:**
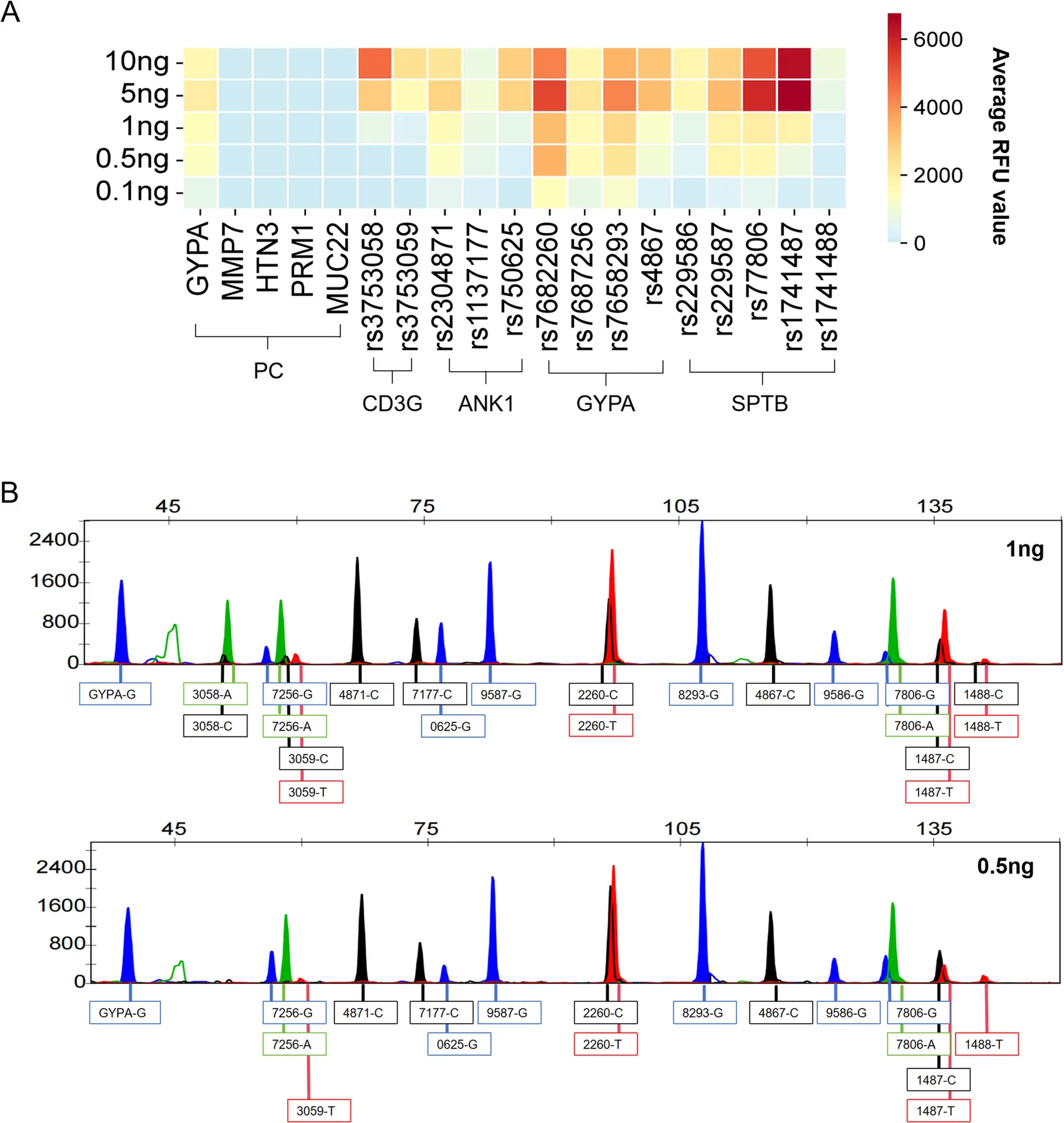
Sensitivity of 14 coding-region single nucleotide polymorphisms (cSNPs) in peripheral blood samples with varying RNA inputs using the SNaPshot assay (*n* = 3). **A** Fluorescence intensity of 14 cSNPs under different RNA inputs (10, 5, 1, 0.5, and 0.1 ng). Fluorescence values are averaged from the three samples, with blue indicating undetected markers. **B** Genotypes of 14 cSNPs using 1 and 0.5 ng RNA templates

The results showed that the fluorescence values of cSNP loci varied across different mRNAs, and differences in peak heights were also observed among different loci on the same mRNA amplicon. As the amount of RNA template decreased, the detected fluorescence values of all loci gradually decreased (Fig. 3A). At 1 ng RNA template, complete genotyping of all 14 cSNPs was still achievable (Fig. 3B). At 0.5 ng, genotyping failure rates were 33.3% for rs1741487 and rs750625, 66.7% for rs1741488, and 100% for rs3753058 and rs3753059 (no detectable peaks across all three replicates). At 0.1 ng, the failure rate was 100% for rs3753058, rs3753059, rs229586, rs1741487 and rs1741488, 66.7% for rs750625, and 33.3% for rs7687256 (Table S4). Therefore, the sensitivity of the assay for detecting single PB samples was 1 ng.

### Detection of cSNPs in mixed samples

Biological samples collected from the crime scene are mostly mixed samples. For example, mixed stains of blood and saliva are common in violent crime scenes. To systematically evaluate the efficacy of the assay in identifying PB in mixed samples, this study prepared RNA samples by mixing PB (from different individuals) with saliva, semen, or vaginal secretions at various gradient ratios.

**Fig. 4:**
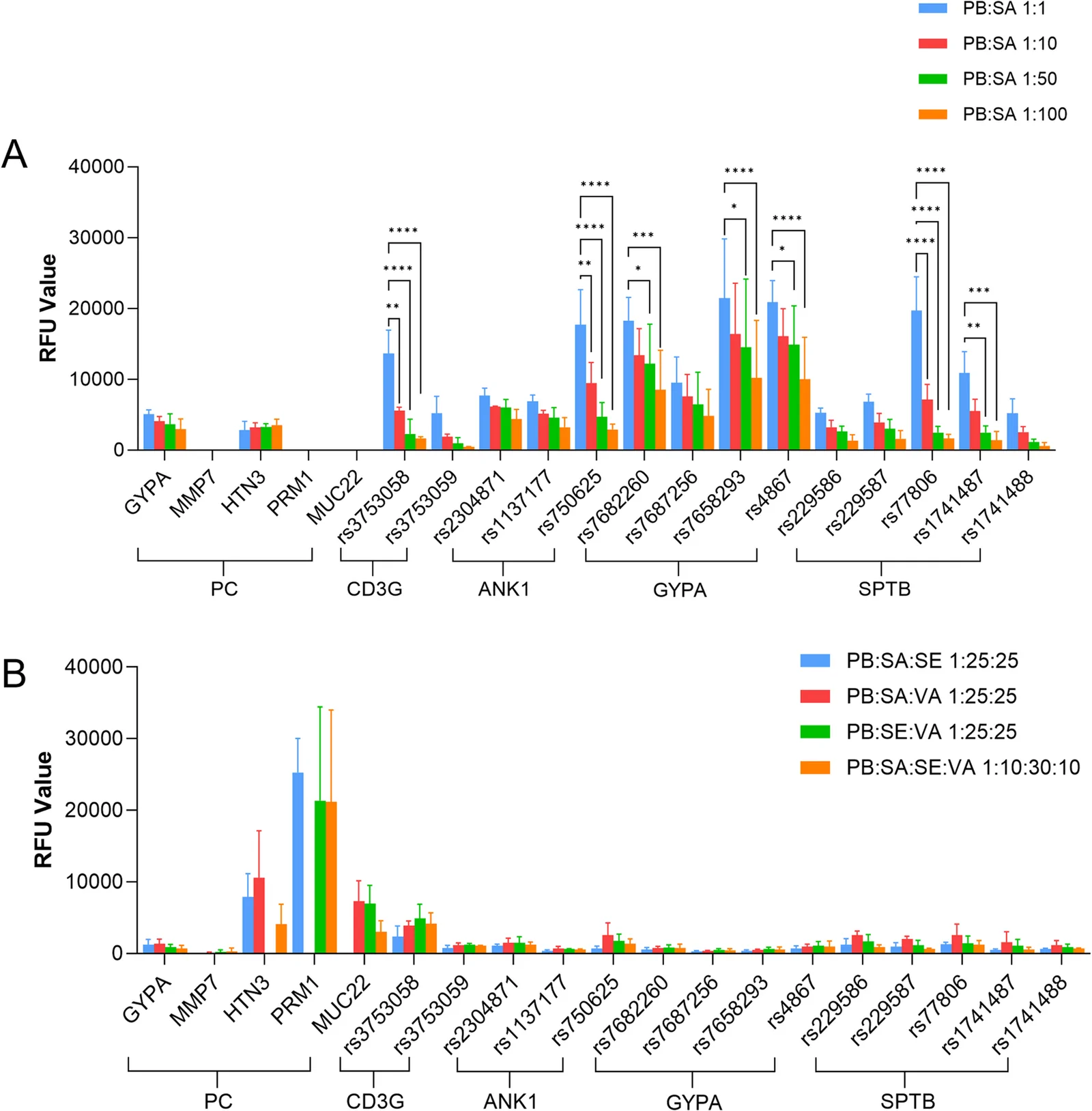
Detection of 14 coding-region single nucleotide polymorphisms (cSNPs) in mixed samples of different body fluids in predefined proportions. **A** Peak values of 14 cSNPs in mixed RNA samples of peripheral blood (PB) and saliva (SA) in various proportions. **B** Peak values of 14 cSNPs in mixed samples containing three to four body fluids in different proportions. The input amount of PB RNA was 5 ng

The results showed that fluorescence signal of each cSNP locus declined gradually as the proportion of PB RNA decreased in mixed body fluids, whereas the peak heights of the positive controls remained unchanged. The 14 cSNPs showed variable detection levels in all pairwise mixed RNA samples (PB-SA, PB-SE, and PB-VA) (Fig. 4A; Figs. S4A and B). Specifically, all 12 cSNP loci were fully genotyped in paired mixed samples. For rs3753059, the genotyping failure rate was 33.3% in PB-SA, PB-SE and PB-VA mixtures at mixing ratios of 1:50 and 1:100. For rs3753058, the genotyping failure rate was 33.3% in PB-SA (1:50) and PB-SE (1:50) mixtures; the rates were 33.3% and 66.7% in PB-SA (1:100) and PB-SE (1:100) mixtures, respectively. With regard to allelic imbalance, a 33.3% imbalance rate was observed for rs7687256 in PB-SA (1:10), PB-SE (1:50) and PB-VA (1:50) mixtures (Table S5). Furthermore, more complex mixed samples containing three or four types of body fluids were constructed, including PB-SA-SE (1:25:25), PB-SA-VA (1:25:25), and PB-SA-SE-VA (1:10:30:10), along with mock case samples of PB-SA (1:1). The 14 cSNPs were also detected in these samples (Fig. 4B), A genotyping failure rate of 33.3% was only observed at rs7687256 in PB-SA-SE-VA (1:10:30:10) mixtures. For rs77806, the allelic imbalance rate was 33.3% in three multi-component mixtures, and reached 66.7% in the PB: SA: VA (1:25:25) mixture (Table S5). These results confirm that the assay has ability to detect PB in mixed samples.

### Comparison of amplification efficiency between one-step and two-step PCR

In previous studies, when detecting cSNP loci on RNA using SNaPshot minisequencing technology, RNA was first reverse-transcribed into cDNA, followed by multiplex PCR amplification, product purification, SBE, and finally CE analysis. In this study, a one-step multiplex RT-PCR amplification system was established, which combines reverse transcription and PCR amplification. This allows reverse transcriptase and DNA polymerase to act sequentially in the same tube under the same buffer conditions, completing the two-step process (reverse transcription and PCR amplification of 4 PB mRNA markers) in a single tube. The performance of one-step multiplex RT-PCR amplification was verified using mixed peripheral blood–saliva samples.

**Fig. 5:**
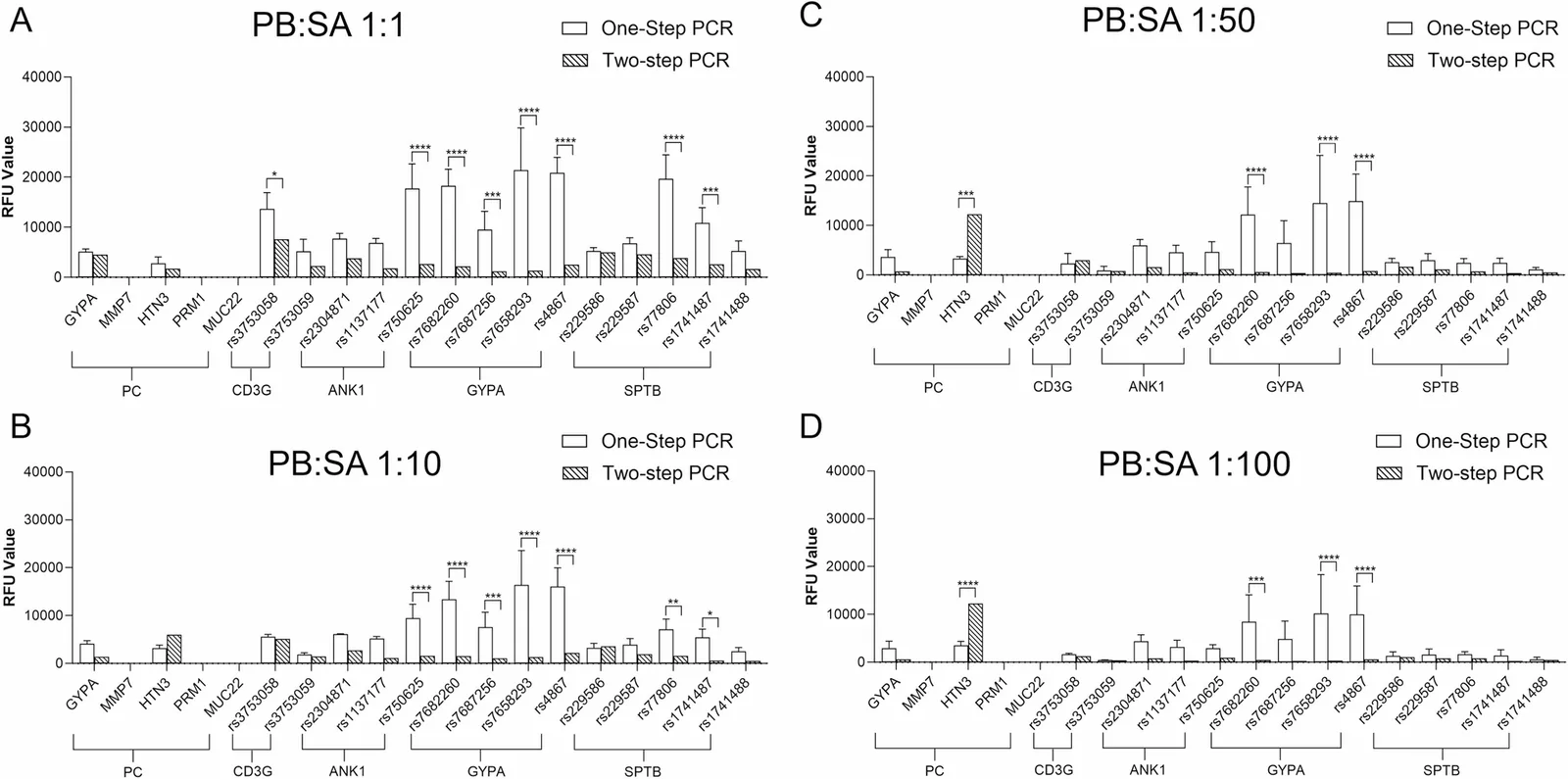
Comparison of the amplification efficiency between the one-step and two-step PCR. **A** and (**B**) display the peak values of 14 coding-region single nucleotide polymorphisms (cSNPs) in peripheral blood (PB) and saliva (SA) RNA samples mixed at a ratio of 1:1 and 1:10, respectively. **C** and (**D**) display the peak values of 14 cSNPs in PB and SA RNA samples mixed at a ratio of 1:50 and 1:100, respectively. ^*^indicates *P* ≤ 0.05, and ^****^indicates *P* ≤ 0.0001

The results showed that in peripheral blood–saliva mixed samples (1:1/1:10), all 14 cSNP loci were effectively detected using the minisequencing assay with one-step multiplex RT-PCR amplification. The fluorescence values of more than half of the cSNP loci were significantly higher than those obtained with the two-step multiplex RT-PCR (Figs. 5A and B). In the 1:50/1:100 mixed samples, the fluorescence values of three cSNP loci were significantly higher than those obtained using the two-step method. Additionally, the fluorescence value of the saliva positive control in the minisequencing assay with two-step multiplex RT-PCR amplification was significantly higher than that in the one-step multiplex RT-PCR system (Figs. 5C and D).

These results indicate that the one-step multiplex RT-PCR amplification established before the SBE reaction can enrich more target PCR product fragments. This not only reduces the operational workflow and minimizes human contamination but also significantly improves the detection sensitivity and stability of the assay for PB in complex mixed samples. The original data can be found in Table S6.

### Consistency between genotyping at the gDNA and RNA levels

**Fig. 6:**
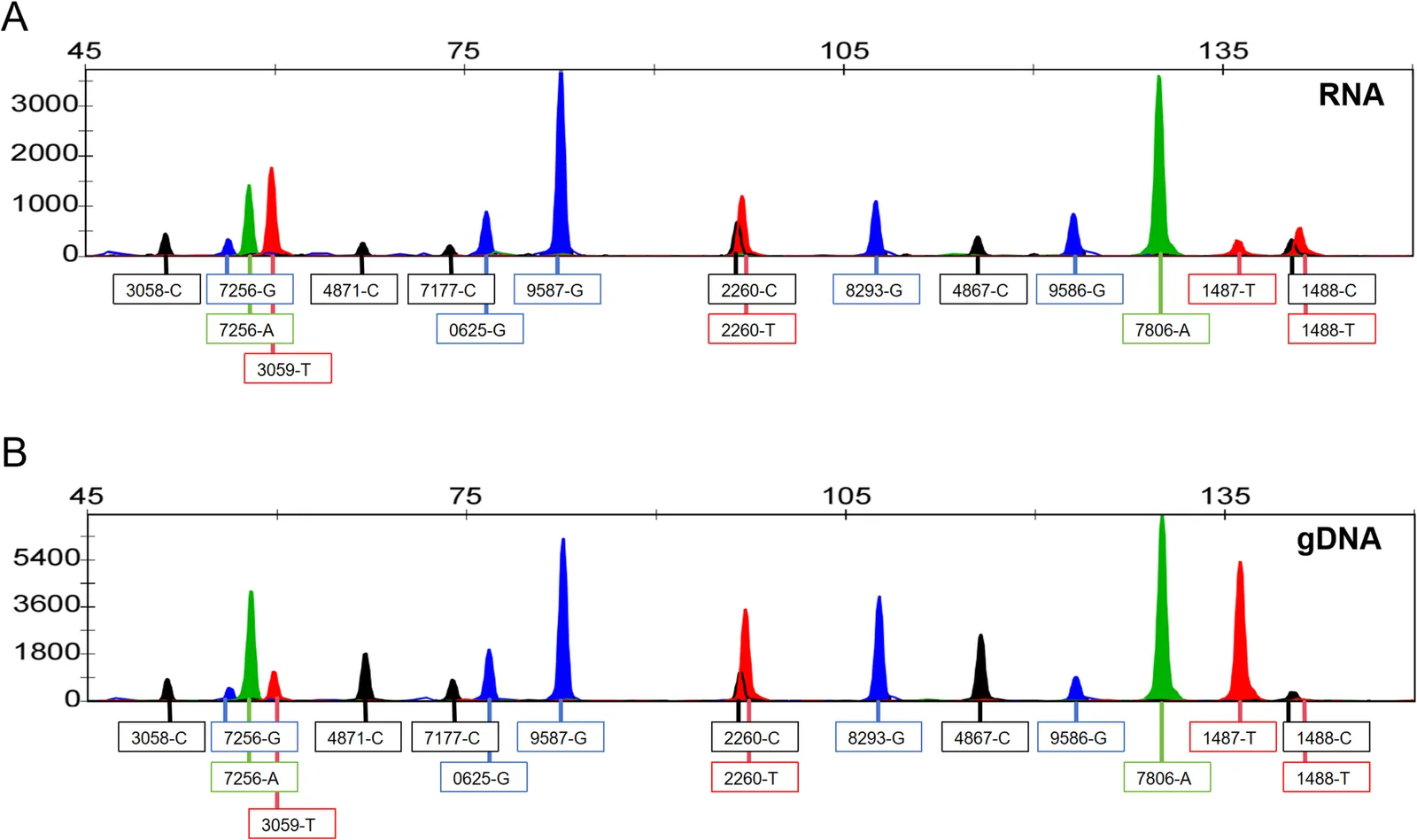
Comparison of genotyping consistency of 14 coding-region single nucleotide polymorphisms (cSNPs) in peripheral blood. **A** RNA template and (**B**) genomic DNA (gDNA) template (*n* = 19)

To ensure that the polymorphism of 14 cSNP loci on peripheral blood gene transcripts can accurately represent those in the genome, a multiplex SNaPshot assay for DNA-cSNPs was also established in this study. Nucleic acids were coextracted from 19 randomly selected peripheral blood samples, and genotyping of the 14 cSNP loci was performed at both DNA and RNA levels. The results showed complete consistency in cSNP genotyping between RNA and DNA across all 19 samples (Fig. 6, Table S7).

### Expression profiling of 14 cSNP loci in large samples

**Fig. 7:**
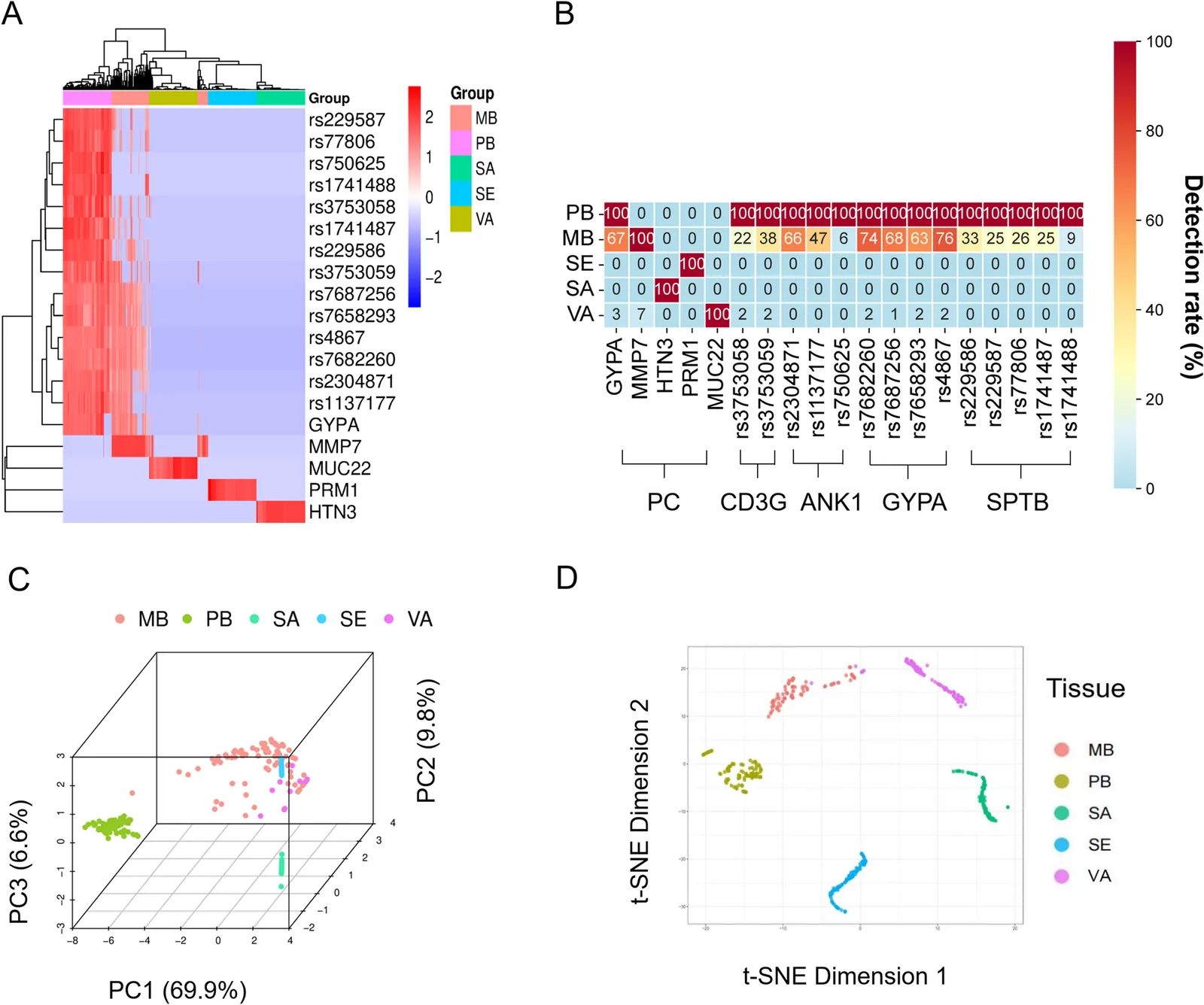
Expression patterns and discrimination capacity of 14 coding-region single nucleotide polymorphisms (cSNPs) in five body fluids. **A** Heat map of 14 cSNPs in five body fluids, with high-expression cSNPs highlighted in red. The x-axis shows the type of body fluid, whereas the y-axis indicates cSNP loci and positive control. **B** Detection rate of 14 cSNPs in five body fluids. **C** and (**D**) t-distributed stochastic neighbor embedding (t-SNE) plots and principal component analysis (PCA) of 14 cSNPs in five body fluid samples (*n* = 100 samples for each type of body fluid)

In this study, a total of 500 body fluid samples from unrelated Han Chinese individuals in northern China were included (100 samples each of PB, menstrual blood, semen, saliva, and vaginal secretions) to evaluate the repeatability and specificity of the assay. The results showed that genotyping of all 14 cSNP loci was stably detected in PB samples (Table S8). In saliva, semen, and vaginal secretions, only the peaks of the corresponding positive controls were detected, with no signals from peripheral blood loci. In menstrual blood samples, 14 cSNP loci were detected at varying degrees (Fig. 7A). Specifically, the positive control locus *GYPA* had a detection rate of 76% in menstrual blood samples. The detection rates of *ANK1*(rs2304871) and *GYPA* (rs7682260, rs7687256, rs7658293, and rs4867) in menstrual blood samples all exceeded 50%. Additionally, sporadic detection of *CD3G* (rs3753058, rs3753059) and *GYPA* (rs7682260, rs7687256, rs7658293, and rs4867) was observed in vaginal secretions (Fig. 7B), suggesting that these loci have an extremely low level of cross-expression.

To preliminary evaluate the overall distribution and batch effect of the samples, we performed dimensional reduction analysis on the fluorescence values of 14 loci across 500 samples. PCA and t-SNE plots were constructed using the logarithm of peak height values based on 14 cSNP markers and five positive controls, and 500 samples were divided into five groups. PCA results showed that the first two principal components (PC1/PC2) explained 79.7% of the total variance. PC1 (69.9%) was mainly associated with body fluid type, whereas PC2 (9.8%) reflected interindividual differences. PB samples showed distinct separation along the first principal component, forming a cluster significantly distinct from the saliva, semen, and vaginal secretion samples (Fig. 7C). We used t-SNE (perplexity = 30, 1000 iterations) to perform nonlinear dimensional reduction analysis on the same dataset and further validated the above results on the local structure. The t-SNE plot showed that samples of each body fluid type formed clusters with clear boundaries in the two-dimensional space (Fig. 7D). Collectively, these results demonstrate that the assay has high specificity and robust discriminative ability for peripheral blood signals.

### Forensic parameters for the 14 cSNP loci

Statistical analysis was performed on the genotyping data of the 14 cSNPs in 100 PB samples from unrelated Han Chinese individuals in northern China. The linkage disequilibrium analysis showed that, except for rs750625, rs1137177, and rs2304871 on the *ANK1* gene, other loci located on the same chromosome were in strong linkage equilibrium, whereas loci on different chromosomes were in linkage equilibrium (Table S9). Loci in strong linkage disequilibrium were treated as haplotypes: haplotype M1 included rs3753059 and rs3753058 on *CD3G*; haplotype M2 included rs1741488, rs1741487, rs77806, rs229587, and rs229586 on *SPTB*; and haplotype M3 included rs4867, rs7687256, and rs7682260 on *GYPA*. The allelic distributions and forensic parameters of loci M1, M2, M3, rs750625, rs1137177, and rs2304871 in the Northern Han Chinese population are presented in Table 4. A total of 2, 11, and 3 haplotypes were observed at the M1, M2, and M3 loci, with corresponding discrimination power values of 0.6112, 0.8512, and 0.4680, respectively. The combined identification efficiency was significantly higher than that of a single locus (Table S10).

**Table 4:** Single nucleotide polymorphism loci and haplotype forensic parameters

Locus	haplotype	*N*	*N*_all_ (number of alleles)	HE	PIC	PM	DP	Ho	PE	*P* value
rs3753059	M1	200	2.0000	0.4993	0.3734	0.3888	0.6112	0.5200	0.2056	0.6927
rs3753058										
rs1741488	M2	200	11.0000	0.6731	0.6435	0.1488	0.8512	0.6700	0.3834	0.0651
rs1741487										
rs77806										
rs229587										
rs229586										
rs4867	M3	200	3.0000	0.4947	0.3971	0.5320	0.4680	0.7400	0.4928	0.0010
rs7687256										
rs7682260										
rs750625	/	200	2	0.1563	0.1435	0.7282	0.2718	0.1500	0.0176	0.5198
rs1137177	/	200	2	0.2492	0.2172	0.6014	0.3986	0.2300	0.0385	0.4203
rs2304871	/	200	2	0.2046	0.1828	0.6698	0.3302	0.1700	0.0221	0.1131

*HE* Expected heterozygosity, *PIC* Polymorphism information content, *PM* Probability matching, *DP* Discrimination power, *Ho* Observed heterozygosity, *PE* Probability of exclusion

“N” denotes the number of alleles, and “/” indicates the absence of a corresponding haplotype

The analysis of forensic parameters for each locus showed that the M3 locus showed a significant deviation from HWE after Bonferroni multiple test correction, which violated the assumption of population genetic equilibrium. To ensure the statistical reliability of forensic parameter estimation, the deviated M3 locus was excluded from the cumulative individual discrimination probability analysis. The CDP of M1, M2, rs750625, rs1137177, and rs2304871 in the Northern Han Chinese population was 0.98303.

## Discussion

In recent years, studies have demonstrated that genetic markers located in DNA coding regions with body fluid specificity, such as cSNPs and insertion/deletion polymorphisms (InDels), can not only identify the source of common body fluid stains, but also retrieve individual genetic information of stain donors, which is particularly valuable for the deconvolution of mixed biological stains [22–24]. This concept was first implemented on the Illumina MiSeq™ platform, where a detection panel was constructed by screening 35 cSNPs from blood, semen, saliva, vaginal secretions, menstrual blood, and skin and its capacity to associate forensic body fluid stains with individual donors was further evaluated [16]. In recent years, several research teams have developed cSNP assays using CE [17, 25, 26] and next-generation sequencing (NGS) platforms [10, 19, 27, 28], aiming to resolve the identification of body fluid stains and their donors. The detection sensitivity of SNaPshot minisequencing assays for body fluids—PB, 16-plex cSNP assay; semen, 12-plex cSNP assay; and vaginal secretions, 18-plex cSNP assay —using a CE platform reached 0.08–1.00 ng. The target body fluids were successfully detected in mixed samples with different proportions, and the consistency of the cSNP markers at the DNA and RNA levels was verified. Considering the linkage disequilibrium, the CDP of PB cSNP markers reached up to 0.99929. In 2020, EUROFORGEN/EDNAP collaborated with nine laboratories to assess the body fluid identification and donor profiling performance of a 35-plex cSNP panel [16], which targets PB, menstrual blood, semen, saliva, vaginal secretions, and skin, using the MiSeq/FGx platform. The results indicated that cSNPs can provide valuable information for associating body fluids or tissues with donors, especially in mixed body fluid stains. However, the amplification of certain cDNAs cannot rule out gDNA interference, and the detection rate of some cSNPs is relatively low, rendering result interpretation challenging [29]. In 2025, EDNAP organized two joint trials involving 11 laboratories, each of which independently selected either the Ion Torrent S5™ or the Illumina MiSeq™ platform to assess the detection and analytical capabilities of two assays — the BFID-cSNP-BSS assay (a 21-plex cSNP assay for blood, saliva, and semen) and BFID-cSNP-6 F assay (a 23-plex cSNP assay for six fluids and tissues, including blood, saliva, semen, vaginal secretions, menstrual blood, and skin)—using simulated casework samples. However, whether based on first-generation CE platforms or NGS platforms, the application of cSNP assays in real cases still raises the following issues worthy of discussion: (I) marker cross-reactivity: few existing cSNPs exhibit absolute body fluid specificity. Cross-expression limits the number of usable loci, and the cumulative individual identification efficacy needs further improvement; (II) population differences: forensic genetic parameters of individual cSNP markers vary across populations from different regions, requiring systematic evaluation of their cross-population discriminatory ability; (III) concordance of cSNP genotyping between genomic DNA and transcribed RNA: Large-scale sample testing is still required to verify how consistently genomic cSNP genotypes are inherited at the RNA transcript level; (IV) platform consistency: CE and NGS differ significantly in detection sensitivity, accuracy, and reproducibility. The comparability of results across laboratories must be strengthened through standardized procedures and quality control systems.

In this study, 14 peripheral blood cSNP loci were screened and identified, and a multiplex SNaPshot minisequencing assay was established. The PB identification performance of this assay were further evaluated. In terms of the detection platform, the SNaPshot assay uses CE, which is the most widely used technique for forensic DNA analysis. This multiplex detection technology offers the advantage of seamless integration into existing forensic DNA analysis workflows without the need for additional equipment. Moreover, SNP panels used in SNaPshot assays can be integrated into custom panels for massive parallel sequencing [30]. Unlike forensic SNP analysis, cSNP analysis requires the reverse transcription of RNA into complementary DNA (cDNA) before subsequent procedures, such as PCR amplification and SBE. The traditional two-step RT-PCR method is time-consuming and operationally cumbersome, and repeated tube opening increases the risk of human contamination. A previous study reported a single-tube multiplex RT-PCR [31], in which reverse transcriptase and DNA polymerase were added to a single tube to complete both reverse transcription and PCR amplification. This method has been widely applied in RNA molecular analysis [32–34]. The F18plex one-step RT-PCR identification system was established for the identification of PB, menstrual blood, vaginal secretion, saliva, semen, and urine [35]. A comparative study of commercial one-step RT-PCR kits and the two-step RT-PCR method regarding their sensitivity and specificity in body fluid stain identification confirmed that the one-step RT-PCR method is suitable for the examination of forensic body fluid samples [36]. In this study, the one-step RT-PCR method was pioneered for integration into the SNaPshot detection assay. The sensitivity of this optimized assay for single PB samples reached 1 ng, and all 14 cSNP loci were effectively detected in mixed peripheral blood–saliva samples in various proportions. The detection peak was significantly higher than those obtained using the traditional two-step method. Although this strategy provides a favorable technical approach for shortening detection time, reducing labor costs, and minimizing artificial contamination, forensic casework samples comprise mostly trace, degraded, and mixed samples, and the detection performance of the newly constructed panel in challenging forensic samples remains to be further verified and optimized.

Regarding the specificity detection of PB markers, cross-reactivity of the same marker across different body fluids increases the difficulty of interpreting identification results; thus, the specificity of the marker molecules requires focused attention. In this study, 11 PB mRNA biomarkers were initially selected through extensive literature research and database retrieval via the Ensembl Genome Browser. However, some mRNAs, such as *HBB*, *HBA*, and *ALAS2*, were excluded due to high-expression levels, low allele frequencies, or the inability to design appropriate primers. Multiple pairs of PCR amplification primers and SBE primers were designed for the eligible mRNA genes and their corresponding cSNP loci. The candidate genes were validated one by one using dry and wet lab tests, discarding mRNA markers and primers with poor specificity and amplification efficiency. Ultimately, 14 cSNP loci located on four PB mRNA molecules, *CD3G*, *ANK1*, *SPTB*, and *GYPA*, were determined. Additionally, nonpolymorphic nucleotides from menstrual blood, saliva, semen, and vaginal secretions were used as positive controls to construct the SNaPshot assay. The results of 500 samples showed that none of the 14 cSNP loci were expressed in saliva or semen. Nevertheless, a total of five cSNP loci on the *ANK1* and *GYPA* genes were detected in more than half of the menstrual blood samples, and the remaining nine loci were detected in menstrual blood samples to varying degrees but with lower peak values than in PB samples, which is related to the fact that menstrual blood samples themselves contain PB components. In addition, a total of 6 loci on *CD3G* and *GYPA* were detected at low levels in individual vaginal secretions, which is consistent with previous reports [17, 19]. Among them, *CD3G* is a T-cell–associated marker; the withdrawal of progesterone before menstruation would eliminate the inhibitory effect, which would lead to the accumulation of leukocytes in the endometrial mesenchyme and therefore *CD3G* may be detected in vaginal secretions [37]. Both PCA and t-SNE analysis demonstrated that the 14 cSNP loci possessed strong discrimination power for PB. Moreover, the system exhibited robust PB identification capabilities in mixed body fluid samples containing two to four components in varying ratios. However, in forensic casework samples, mixed stains consisting of peripheral blood, menstrual blood and vaginal secretions are frequently encountered, especially in sexual assault cases. Theoretically, with a sufficient number of markers, cSNPs can provide a reference for body fluid identification and donor individualization of mixed stains. Nevertheless, the present panel still falls short of this practical application goal, and further validation and systematic evaluation using large-scale mixed sample sets are still required.

Theoretically, cSNPs transmit polymorphic information from DNA to RNA. However, inconsistencies between DNA and RNA genotyping have been observed in practical detection. For instance, heterozygous genotypes at the DNA level of genes such as *TGM4*, *KLK4*, and *MUC22* were detected as homozygous at the RNA level [27, 38]. In this study, we established parallel DNA-cSNP and RNA-cSNP assays and randomly tested 19 PB samples for consistency across the 14 loci. The results showed complete agreement between the polymorphisms detected at the RNA and DNA levels. However, gene expression is modulated by multiple genetic regulatory factors, which may compromise the cSNP genotypic concordance in transcripts. Furthermore, RNA degradation and variable reverse transcription efficiency can reduce DNA-RNA genotyping consistency. Accordingly, the DNA–RNA genotypic consistency of these cSNPs needs further validation using larger sample cohorts and degraded forensic samples. Based on this, the present study analyzed the linkage disequilibrium status and inferred the putative haplotypes of the 14 cSNP loci, followed by an evaluation of their individual identification efficacy. In this study, PHASE software was used to infer the potential haplotypes of the M1, M2, and M3 loci. The results showed that the M2 locus, encompassing rs1741488, rs1741487, rs77806, rs229587, and rs229586 within the SPTB gene, exhibited favorable individual identification capacity. In contrast, the M3 locus deviated from HWE in the Northern Han Chinese population, which may be attributed to population substructure and stratification. Furthermore, the haplotypes of the M3 locus were not determined by direct allele combination typing but were reconstructed using PHASE software, which may also partially account for the HWE deviation of this locus. As noted in previous reports, SNaPshot detection cannot determine haplotype phases when two or more SNP loci are simultaneously heterozygous, whereas massive parallel sequencing overcomes these technical limitations and has become a preferred analytical platform for related research in forensic laboratories worldwide [17, 39].

## Conclusions

In this study, a highly sensitive 14-plex cSNP SNaPshot minisequencing assay for PB was established, and the results of large-scale sample testing demonstrated that the assay could stably identify PB components in both single and mixed body fluid samples, with a detection sensitivity of 1 ng. However, given the inevitable presence of PB components in menstrual blood, this detection system cannot yet serve as an accurate identification method for mixed stains of PB and menstrual blood in practical applications. In addition, the selected loci were consistent at both the DNA and RNA levels. The CDP of this panel reached 0.98303 when tested in 100 unrelated Han Chinese individuals. This system provides a reliable technical approach and data support for addressing the forensic identification of PB. With the continuous advancement of RNA sequencing technology, more novel body fluid RNA markers, including microRNAs, PIWI-interacting RNAs, and circular RNAs, are constantly being identified and validated. This will further expand the number of cSNP loci to enhance individual discrimination power and broaden the applicability of this system in simulated casework samples.

## Supplementary Information

Supplementary Material 1. Fig. S1: Results of the specificity validation experiment for mRNA primers for peripheral blood samples and positive controls. (A) The primer specificity of 8 peripheral blood-associated mRNA amplicons and five body fluid-associated mRNA amplicons was verified by electronic PCR. (B) The primer specificity was verified by agarose gel electrophoresis. Fig. S2: Sensitivity of 14 coding-region single nucleotide polymorphisms (cSNPs) in peripheral blood samples with varying RNA inputs using the SNaPshot assay (*n* = 3). (A) and (B) Genotypes of 14 cSNPs using 10 and 5 ng RNA template, respectively. Fig. S3: Detection of 14 coding-region single nucleotide polymorphisms (cSNPs) in mixed RNA samples from different body fluids in predefined proportions. (A) and (B) respectively illustrate the genotypes of 14 cSNPs in mixed samples of peripheral blood (PB) and saliva (SA) in a 1:1 ratio and PB and semen (SE) in a 1:1 ratio. Fig. S4: Detection of 14 coding-region single nucleotide polymorphisms (cSNPs) in mixed RNA samples from different body fluids in predefined proportions. (A) and (B) respectively show the peak values of 14 cSNPs in mixed samples of peripheral blood (PB) and semen (SE) and PB and vaginal secretions (VA) in various proportions.

Supplementary Material 2.

Supplementary Material 3.

## Data Availability

All data needed to evaluate the conclusions in the paper are presented in the paper and associated supplementary materials. Additional datasets that support the findings of this study are available from the corresponding author upon reasonable request. The data are not publicly available due to privacy and ethical restrictions.

## Abbreviations

cSNP: Coding-region single nucleotide polymorphism
RT-PCR: Reverse transcription PCR
CDP: Cumulative discrimination power
STR: Short tandem repeat
SNP: Single nucleotide polymorphism
EDNAP: European DNA Profiling Group
CE: Capillary electrophoresis
NGS: Next-generation sequencing
gDNA: Genomic DNA
SBE: Single-base extension
PCA: Principal component analysis
t-SNE: t-distributed stochastic neighbor embedding
HWE: Hardy–Weinberg equilibrium
cDNA: Complementary DNA
