# Supplementary material for: High-efficiency 14-plex cSNP SNaPshot assay for forensic peripheral blood identification
Source: BMC Genomics. 2026 Jul 11;27:821. doi: 10.1186/s12864-026-13173-0 (PMC13644189; doi:10.1186/s12864-026-13173-0)
Supplement: Supplementary file 1 [file 12864_2026_13173_MOESM3_ESM.pdf]

All information compliant with the MIQE guidelines for PCR experiments performed in this study is summarized in the table below.

Category	Item of MIQE guideline	The content of this study involves the items
Experimental design	• Definition of experimental and control groups	• Experimental group consisted of RNA extracted from healthy body fluid specimens to be tested; controls included no-template control (NTC) and no-reverse-transcription control (-RT control).
Sample	• Number of biological replicates per group	• Three biological replicates plus one no-template negative control were included for each group.
	• Sample description and processing procedures	• See the section "Sample collection and storage"
	• Initial sample volume/mass used for processing	• See the section "Nucleic acid extraction and quantification"
Nucleic acid extraction	• Extraction protocol and instrumentation<br>• Extraction kit information and any protocol modifications<br>• Details of DNase/RNase treatment	• See the section "Nucleic acid extraction and quantification"
	• Nucleic acid contamination assessment (DNA/RNA)	• No-reverse-transcription controls were included in the two-step RNA detection, and no-template controls were set up during PCR amplification to monitor contamination.
	• Quantification platform and method<br>• RNA integrity assessment (method/instrument)	• See the section "Nucleic acid extraction and quantification"
Primer	• Primer sequences and working concentrations	• Supplementary Tables 2 and 3
	• Site and type of primer modification	• Single-base extension (SBE) primers were modified at the 5' terminal modifications.<br>See the section "cSNP selection and primer design"
Reverse transcription	• Full reaction conditions for reverse transcription and subsequent amplification	• See the section "Two-step multiplex RT-PCR amplification"
	• Input RNA quantity and total reaction volume	• See the section "Two-step multiplex RT-PCR amplification" and "One-step multiplex RT-PCR amplification"
	• Reverse transcriptase source and concentration<br>• RT incubation temperature and duration	• See the section "Two-step multiplex RT-PCR amplification"
Target information	• Gene selection and primer design strategy	• See the section "cSNP selection and primer design"
Data analysis	• Number and consistency of biological replicates	• Three consistent biological replicates were performed
	Number and experimental stage of technical replicates (RT or amplification)	• Three technical replicates were arranged at the amplification detection stage
	• Statistical methods for significance analysis	• See the section "Statistical analysis"

• Analysis software (source & version)	• GeneMapper™ ID-X software v1.5

Explanation for MIQE items not applicable to the current study：

The present work employed an endpoint multiplex RT-PCR combined with SNaPshot genotyping workflow for gene variation/genotyping detection instead of quantitative real-time PCR (qPCR). Accordingly, MIQE criteria related to absolute or relative gene quantification are not applicable here, detailed as follows:

1. Standard curve and amplification efficiency: No calculation of template copy number or relative gene expression was performed; hence standard curve construction, amplification efficiency calculation and R^2 evaluation were not required.

2. Cq/Ct value and threshold setting: All tests were analysed at the endpoint of PCR rather than real-time monitoring, so no amplification curves, Cq values or fluorescence threshold settings were generated.

3. Melting curve analysis: Amplicon specificity was validated via fragment size and distinct fluorescent labeling of SNaPshot extension products; melting curve testing was unnecessary.

4. Normalization using reference genes: This study focused on endpoint genotyping rather than quantitative expression analysis, and normalization against reference gene Cq values was not implemented.

5. Limit of quantification: Only limit of detection (LOD) was determined instead of lower limit of quantification (LLOQ), as precise quantitative measurement was beyond the scope of this assay.

6. Reverse transcription efficiency: Adequate yield of cDNA was sufficient for downstream genotyping, and independent quantification of reverse transcription efficiency was not required.

All remaining MIQE items concerning experimental quality control have been reported as summarized above. Collectively, the reliability and reproducibility of the developed assay are fully guaranteed.
